# Linezolid Toxicity: A Clinical Case Report

**DOI:** 10.7759/cureus.55672

**Published:** 2024-03-06

**Authors:** Ângela Ferreira, Patrícia Sobrosa, Miguel Costa, Irene Miranda, Diana Guerra

**Affiliations:** 1 Internal Medicine, Hospital de Santa Luzia-Unidade Local de Saúde do Alto Minho, Viana do Castelo, PRT

**Keywords:** lactic acidosis, serotonine syndrome, drug toxicity, drug interactions, linezolid

## Abstract

Linezolid plays a clinically important role; however, it is responsible for severe pharmacological interactions and side effects, such as myelosuppression, serotonin syndrome, and lactic acidosis. We report a case of an 80-year-old man treated with venlafaxine for depression. He was admitted with a right femur fracture and submitted to surgical intervention, complicated by local infection. In collected pus was identified multiple microorganisms including *Enterococcus faecium* resistant to vancomycin. The therapeutic was adjusted to linezolid. On the 36^th^ day of treatment, he developed hypertension, poor peripheral perfusion, and generalized tremor. He was disoriented, with marbled skin, myoclonus, and sinus tachycardia; and apyretic, with no signs of respiratory distress or joint/surgical wound inflammatory signs. Blood tests showed hyperlacticemia and discrete elevation of C-reactive protein but in a decrescent trend, with no other relevant alterations. The diagnosis of lactic acidosis and probable serotonin syndrome secondary to linezolid was made, supported by improvement after the drug suspension.

## Introduction

Linezolid is an antimicrobial agent with bacteriostatic activity against gram-positive microorganisms, inhibiting the initiation of bacterial protein synthesis at the 50S ribosome subunit [1]. Its action against methicillin-resistant and vancomycin-resistant agents is clinically important [1-3]. Currently, it has been used in the treatment of bone and joint infections, orthopedic implant infections, and multidrug-resistant tuberculosis [4].

The recommended maximum duration of treatment with this antibiotic is 28 days, but it has been used for longer periods, such as in orthopedic implant infections (usually three months [5], but it could be different if the patient is treated with or without removal of the orthopedic implant [4,6]).

The most significant side effects of this antimicrobial agent are gastrointestinal symptoms; myelosuppression, with a higher risk when there is a pre-existing cytopenia, renal insufficiency, and a duration of antimicrobial therapy > 14 days; peripheral or optic neuropathy, with no description of the dose or treatment duration that provoke this toxicity, and the mechanism of neuronal damage is uncertain (in the case of this side effect, the antibiotic should be discontinued); lactic acidosis that occurs due to mitochondrial toxicity, usually happens in situations of prolonged use (≥ 28 days), and should be discontinued immediately due to its high associated mortality (25.5%) [1-3,7].

Linezolid is also a nonselective inhibitor of monoamine oxidase (MAO) A and B, which can lead to increased activity of other drugs such as MAO inhibitors and serotoninergic and adrenergic agents, increasing the risk of serotonin toxicity when used concurrently [1,2]. Pharmacological interactions are also described with various drugs, such as meperidine, fentanyl, heophylline, epinephrine, norepinephrine, escitalopram, dopamine, albuterol, tramadol, metformin, risperidone, haloperidol, aripiprazole, granisetron, trimethoprim/sulfamethoxazole, quetiapine, and metoclopramide [2].

Therefore, despite being an important antimicrobial agent for the treatment of infections caused by resistant microorganisms, it can be responsible for serious pharmacological interactions [2].

This article was previously presented as an oral communication at the 27^th^ National Congress of Internal Medicine, at the Algarve Congress Center-Portugal, on October 4, 2021.

## Case presentation

This is the case of an 80-year-old man with hypertension, type 2 diabetes mellitus, chronic obstructive pulmonary disease, depression, and a total right hip prosthesis. He was regularly medicated with antihypertensive drugs, antidiabetics (metformin 1000 mg and gliclazide 60 mg, which were discontinued upon admission), and 150 mg of venlafaxine. He had no known drug allergies.

**Figure 1:**
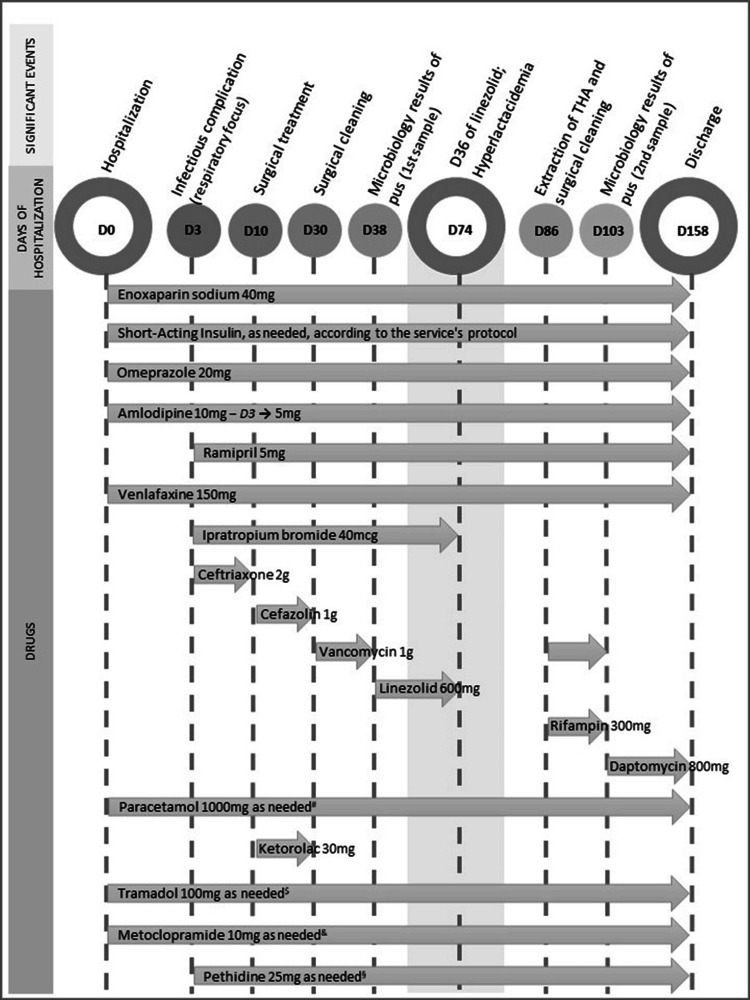
Medication history throughout hospitalization THA: Total hip arthroplasty; ^#^–daily doses or every two days; ^$^–four doses between D1 and D59: dose on D12, D30, D31, and D32; four doses between D97 and D103: dose on D97, D98, D100, and D103; ^&^–five doses between D1 and D59: dose on D12, D28, D30, D31, and D32; five doses between D82 and D103: dose on D82, D97, D98, D100, and D103; ^§^–dose on D10 and D41

He was admitted after a fall with a traumatic brain injury and a pelvic fracture with a periprosthetic fracture of the right femur. He was hospitalized for surgical treatment, receiving analgesia, antihypertensive drugs, insulin, and venlafaxine (Figure 1).

On the 10^th^ day of hospitalization, he underwent surgical correction, complicated by local infection and the need for surgical cleaning with intraoperative pus collection. On that date, blood cultures were negative (Table 1).

**Table 1:** Microbiology results

Days of hospitalization	30	84	86
	Under cefazolin	Seven days after discontinuation of linezolid	Nine days after discontinuation of linezolid
Blood cultures		Two samples for aerobic microorganisms	
		Negative	
		Two samples for anaerobic microorganisms	
		Negative	
Pus	Enterococcus faecalis		Enterococcus faecium
	Sensitive to ampicillin and vancomycin; intermediate sensitivity to imipenem		Sensitive to linezolid; resistant to ampicillin and vancomycin
	Enterococcus faecium		
	Sensitive to linezolid; resistant to ampicillin, imipenem, and vancomycin		
	Staphylococcus epidermidis		
	Sensitive to vancomycin, clindamycin, fusidic acid, gentamicin, moxifloxacin, trimethoprim/sulfamethoxazole; intermediate sensitivity to ciprofloxacin and tetracycline; resistant to erythromycin and oxacillin		

Initially treated with vancomycin, it was adjusted to linezolid after the identification of *Staphylococcus epidermidis*, vancomycin-resistant *Enterococcus faecium* (VRE), and *E. faecalis* in the pus, with an indication to complete 12 weeks.

On the 36^th^ day of linezolid, the patient presented hypertension (200/90 mmHg) and generalized tremors. The orthopedic medical team prescribed captopril 25 mg and requested an evaluation from the internal medicine team. In the medical evaluation, he was vigilant, disoriented in time and space, cooperative, and with myoclonus but without focal neurological deficits or meningeal signs. Blood pressure was 143/80 mmHg, heart rate 120 beats per minute, respiratory rate 20 breaths per minute, oxygen saturation 98% with oxygen supplementation at 1L/minute, no signs of respiratory distress, axillary temperature 37.3°C, capillary perfusion time < 2 seconds, and normoglycemic. He had marbled skin with sweating and paleness and hydrated mucous membranes and was jaundice-free. Pulmonary and cardiac auscultation revealed no abnormalities except for tachycardia. The abdomen was soft and depressible, without tenderness. No peripheral edema and no inflammatory signs in the joints or surgical wound were noted. Electrocardiogram showed sinus tachycardia at 120/minute, with no other alterations. Blood tests (Table 2) showed a slightly increased C-reactive protein but in a decreasing profile, hyperlactacidemia (7.3 mmol/L), normal muscular markers, and no relevant respiratory insufficiency or other alterations.

**Table 2:** Arterial blood gas and laboratory test results ALT: Alanine aminotransferase; AST: aspartate aminotransferase; DB: direct bilirubin; BNP: B-type natriuretic peptide; TB: total bilirubin; ERS: erythrocyte sedimentation rate; ALP: alkaline phosphatase; FiO2: fraction of inspired oxygen; GamaGT: gamma-glutamyl transferase; LDH: lactate dehydrogenase; MCHC: mean corpuscular hemoglobin concentration; MCV: mean corpuscular volume; pCO2: partial pressure of carbon dioxide; pO2: partial pressure of oxygen

Days of hospitalization		74	80	84	109
Significant events		D36 of linezolid	Three days after linezolid suspension	Seven days after linezolid suspension	Seven days after starting daptomycin
Arterial blood gas	FiO_2_	24%	21%		
	pH	7.41	7.48		
	pO_2_	87 mmHg	70 mmHg		
	pCO_2_	44 mmHg	54 mmHg		
	Oxygen saturation	98%	95%		
	HCO_3_^-^	27.9 mmol/L	36.6 mmol/L		
	Lactate	7.3 mmol/L	0.6 mmol/L		
	Ca^2+^	1.16 mmol/L	1.16 mmol/L		
Complete blood count, biochemistry	Hemoglobin	9.2 g/dL	8.3 g/dL	8.1 g/dL	10.5 g/dL
	MCV	90.0 fL	87.2 fL	90.4 fL	91.8 fL
	MCHC	32.1 g/dL	33.1 g/dL	32.9 g/dL	32.4 g/dL
	Leukocytes	4.57x10^9^/L	6.35x10^9^/L	7.14x10^9^/L	8.08 x10^9^/L
	Neutrophilis	90%	73%	73%	75%
	Platelets	173x10^9^/L	139x10^9^/L	243x10^9^/L	386 x10^9^/L
	ERS	45 mm			
	Glucose	181 mg/dL	150 mg/dL	127 mg/dL	170 mg/dL
	Urea	29.0 mg/dL	35.0 mg/dL	33.0 mg/dL	28.0 mg/dL
	Creatinine	0.55 mg/dL	0.46 mg/dL	0.47 mg/dL	0.45 mg/dL
	Na^+^	134 mmol/L	134 mmol/L	139 mmol/L	138 mmol/L
	K^+^	4.1 mmol/L	4.6 mmol/L	4.1 mmol/L	5.1 mmol/L
	TB/DB, ALP, GamaGT, AST/ALT	No alterations			
	LDH	185 UI/L	254 UI/L	179 UI/L	
	C-reactive protein	2.24 mg/dL	4.85 mg/dL	16.03 mg/dL	5.93 mg/dL
	Myoglobin, troponin I, BNP	No alterations			
Urinalysis		No alterations			

The chest X-ray showed cardiomegaly without pleural effusion, pneumothorax, or consolidation. The brain computed tomography scan showed no significant intra- or extra-axial abnormalities, including parenchymal areas that might indicate recent ischemic, hemorrhagic, or contusional events. An electroencephalogram was performed a few days after the event showed no epileptiform activity.

Given the clinical presentation and tests results, the hypothesis of hyperlactacidemia and probable serotonin syndrome as a side effect of linezolid were assumed. The patient had been concurrently medicated with venlafaxine since the beginning of hospitalization, with no recent dose changes or other therapeutic adjustments. Linezolid was suspended on the 39^th^ day of treatment (Figure 1), after being aware of the results of all tests to rule out other causes for the symptoms presented. Three days later (on the 80^th^ day of hospitalization), there was resolution of lactic acidosis (Table 2) and the remaining symptoms.

However, he presented again with an increase in inflammatory parameters (Table 2) and underwent a new surgical intervention for cleaning and debridement. Vancomycin associated with rifampicin was restarted, but the microbiology result of the pus identified VRE sensitive only to linezolid. Antibiotic therapy was adjusted, and daptomycin 10 mg/kg was initiated. Due to a new elevation of inflammatory parameters, prosthesis extraction from the hip was performed. Gradually, the patient showed clinical and analytical improvement (Table 2), at 109 days of hospitalization.

## Discussion

Lactic acidosis is defined by a serum lactate concentration > 4mmol/L, even in the absence of evident acidemia [8]. Causes of lactic acidosis can be divided into various types. Type A is linked to decreased tissue oxygenation, usually due to tissue hypoperfusion resulting from hypovolemia, heart failure, sepsis, or cardiorespiratory arrest. Type B arises from cellular metabolism alterations induced by toxins or the presence of ischemic areas, and it can occur in patients with diabetes mellitus on biguanides, cancer (leukemia, lymphoma, or solid tumors), alcoholism, HIV infection, and the use of reverse transcriptase nucleoside inhibitors, intravenous administration of beta-adrenergic agonists (intravenous epinephrine), mitochondrial dysfunction, or drugs inducing mitochondrial dysfunction [3,8]. There is often an overlap between these two types of lactic acidosis. Type D is a rare form that can occur in patients with short bowel syndrome or other forms of malabsorption [8]. Linezolid is known to cause type B lactic acidosis due to mitochondrial dysfunction [3,8]. In addition to inhibiting the 50S subunit of bacterial ribosomes, it affects the ribosomes of human cell mitochondria and their protein synthesis [8]. It typically appears several weeks after the start of treatment, but cases have been reported that emerged a few days after the introduction of the antibiotic, especially in immunosuppressed patients or those initiating immunosuppressive therapy [3]. In the literature are described cases of linezolid-induced lactic acidosis with nonspecific signs and symptoms, enumerated in Table 3 [4]. Despite being a known side effect, it is a diagnosis of exclusion and can be lethal if not identified promptly [3,4].

**Table 3:** Most common side effects of linezolid Adapted from Santini A, Ronchi D, Garbellini M, Piga D, Protti A. Linezolid-induced lactic acidosis: the thin line between bacterial and mitochondrial ribosomes [4]

The most common side effects of linezolid	
Side effect	Percentage (%)
Nausea and vomiting	33
Changes in mental status	27
Anemia or thrombocytopenia	23
Abdominal pain	19
Dyspnea	19
Asthenia	19
Cardiovascular collapse	13
Tachycardia	10
Diarrhea	8
Liver dysfunction	6
Pancreatitis	4
Hypoglycemia	4
Hypothermia	4

In this case report, the patient had been on linezolid for five weeks when he presented with disorientation, tachycardia, and hyperlactatemia. Other causes of lactic acidosis were ruled out: worsening of the infectious focus under treatment or other foci and no severe anemia or suggestive changes of heart failure, acute coronary syndrome, or liver failure. Given a recently traumatic brain injury, late complications or seizures were ruled out. After excluding these diagnostic hypotheses, linezolid-induced lactic acidosis was assumed, leading to its suspension and subsequent clinical and analytical improvement.

However, the symptoms were not fully explained by lactic acidosis alone. In this context, another cause for the presented symptoms was sought: generalized tremors, myoclonus, and diaphoresis. Upon reviewing the patient's therapy (Figure 1), the presence of drugs administered concurrently with linezolid that increased the risk of serotonin toxicity was noted, namely, the selective serotonin reuptake inhibitor (SSRI) venlafaxine, prescribed since the beginning of hospitalization, as well as salbutamol, tramadol, and metoclopramide, which could also increase serotonin toxicity. However, the latter had not been administered in the two weeks before the presentation of symptoms.

Serotonin syndrome is a potentially fatal condition related to increased serotonin activity in the central nervous system, which can occur through medication use, pharmacological interaction, or voluntary intoxication with serotonergic agents [9]. It presents with a wide variety of signs and symptoms, such as tachycardia, hypertension, hyperthermia, agitation, tremors, myoclonus, hyperreflexia, muscle rigidity, mydriasis, dry mucous membranes, increased bowel sounds, and diaphoresis [1,9,10]. These can appear hours, days, or weeks after the co-administration of linezolid with serotonergic agents [1,9,10]. The diagnosis of serotonin syndrome is clinical, and criteria from Hunter, Sternbach, or Boyer (Table 4) can be used [9,10].

**Table 4:** Criteria for the diagnosis of serotonin syndrome ^#^The Boyer criteria are more specific than the Sternbach criteria [10]

Criteria for diagnosis of serotonin syndrome		
Hunter Criteria [9]	Sternbach Criteria [10]	Boyer Criteria^#^ [10]
Presence of a serotonergic agent plus one of the following criteria:	Presence of at least three of the following criteria, in the absence of the use of neuroleptics or another etiology:	Presence of a serotoninergic agent administered in the previous five weeks and any of the following criteria (already described in Hunter's criteria):
Spontaneous clonic spasm	Altered mental status	Spontaneous clonic spasm
Inducible clonic spasm + agitation or diaphoresis	Agitation	Inducible clonic spasm + agitation or diaphoresis
Ocular clonic spasm + agitation or diaphoresis	Myoclonus	Ocular clonic spasm + agitation or diaphoresis
Tremor + hyperreflexia	Hyperreflexia	Tremor + hyperreflexia
Hypertonia + temperature > 38ºC + Ocular or inducible clonic spasm	Diaphoresis	Muscle rigidity + temperature > 38ºC + inducible or ocular clonic spasm
	Shivering	
	Tremor	
	Diarrhea	
	Discoordination	
	Fever	

In the presented case, the patient was medicated with linezolid and had an indication for a long antibiotic course (12 weeks) due to an infection related to orthopedic implant; on the 36^th^ day of treatment, he presented symptoms that met Sternbach criteria (disorientation, tremors, myoclonus, and diaphoresis) and Boyer/Hunter's criteria (serotonergic drug and spontaneous clonic spasms), making the diagnosis of serotonin syndrome secondary to linezolid likely. Treatment, besides symptom control, includes discontinuation of drugs, with symptom resolution within one to five days [1,9,10]. Due to this risk, discontinuation of serotonergic drugs two weeks before starting linezolid treatment is recommended [1,2]. However, the introduction of the antimicrobial agent should not be delayed for this reason, so monitoring for signs and symptoms of serotonin toxicity is advised [10].

Given the potentially fatal interactions and adverse effects of linezolid, it is important for all patients undergoing prolonged treatment (>seven days) to be monitored weekly with complete blood count, liver function, and lactate levels [1,4]. If treatment exceeds 28 days, regular ophthalmological and neurological evaluations should be performed [1]. In situations where drugs such as selective serotonin reuptake inhibitors (SSRIs) or pro-serotonergic agents cannot be discontinued, monitoring for serotonin toxicity is advised, for at least three weeks or more if antimicrobial therapy continues [1,10]. In the presented case, after discontinuation of linezolid and the need to maintain antimicrobial therapy, daptomycin was chosen, a bactericidal agent approved for the treatment of infections by vancomycin-resistant Enterococcus (VRE) [11,12].

## Conclusions

Therefore, despite its clinical importance, the potentially severe adverse effects of linezolid underscore the need for careful surveillance and monitoring, especially in patients requiring prolonged treatment. The presented case, where the patient developed lactic acidosis and serotonin syndrome, as per casuality assessment induced by linezolid, represents known adverse effects of this drug. In this situation, it was possible to identify lactic acidosis as an adverse effect of the antibiotic administered over an extended period, after ruling out other more common causes. However, the persistence of symptoms and the presence of symptoms that could not be explained solely by lactic acidosis led to the identification of serotonin syndrome, emphasizing the need to review the entire medical history, perform a thorough physical examination, and assess the treatment administered to understand the patient's clinical picture and arrive at a diagnosis.

Furthermore, the decision to discontinue linezolid and the need to maintain antimicrobial therapy, opting for the initiation of daptomycin, highlights the necessity of adapting and making clinical decisions based on the patient's clinical evolution and microbiology results.
